# La métaplasie osteoide de l'endomètre après une grossesse à terme: à propos d'un cas rare

**DOI:** 10.11604/pamj.2013.15.14.2573

**Published:** 2013-05-08

**Authors:** Sofia Jayi, Hakima Bouguern, Hind Fatemi, Hikmat Chaara, Afaf Laamarti, Aabdelilah Melhouf

**Affiliations:** 1 CHU Hassan II de Fes, Maroc; 2 Université sidi mohammed ben abdellah, Maroc

**Keywords:** Métaplasie-ostéoïde, endomètre, grossesse à terme, diagnostic, hystéroscopie, Osteoid metaplasia, endometrium, term pregnancy, diagnosis, hysteroscopy

## Abstract

La métaplasie ostéoïde de l'endomètre (MOE) est une entité rare correspondant à la présence de tissu osseux dans l'endomètre, elle est le plus souvent diagnostiquée dans un contexte d'infertilité secondaire faisant suite à une grossesse interrompue. Même si plusieurs facteurs de risque sont répertoriés, sa physiopathologie reste mal connue et sa traduction clinique est très variable. Nous rapportons un cas de MOE apparu suite à un curetage pour rétention placentaire en post-partum. Le diagnostic a été suspecté par l'hystéroscopie et confirmé par l’étude anatomopathologique. A notre connaissance c'est le premier cas décrit suite à un accouchement à terme. A travers notre cas et à la lumière d'une revue de la littérature nous insistons sur les caractéristiques épidémiologiques, physiopathologiques, cliniques et para cliniques de cette entité rare, dont la connaissance est primordiale pour un diagnostic sûr et par conséquent un traitement adapté permettant souvent de récupérer la fertilité de la patiente.

## Introduction

La métaplasie ostéoïde de l'endomètre (MOE), est une affection rare correspondant à la présence dans l'endomètre de tissu osseux ectopique [1]. Elle a été décrite la première fois en 1901, cependant cette pathologie semble méconnue, insuffisamment cherchée, sous-diagnostiquée [2] et par conséquent mal traitée. Nous rapportons un cas de MOE à travers lequel nous soulignons la difficulté diagnostic et l'intérêt de l'hystéroscopie dans le diagnostic et le traitement.

## Patient et observation

**Figure 1:**
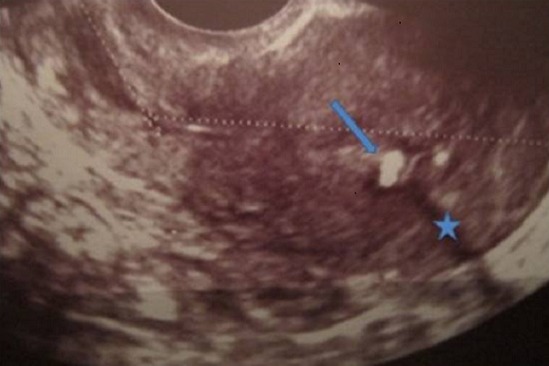
Coupe longitudinale de l'utérus en échographie, montrant une image hyper-échogène (flèche) avec cône d'ombre postérieur (étoile)

**Figure 2:**
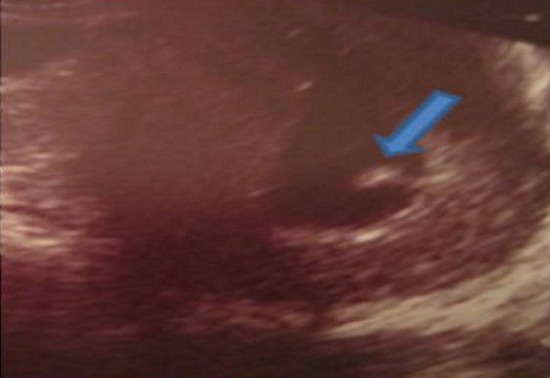
Image hysterosonographique confirmant la localisation intracavitaire de l'image hyperéchogène (flèche)

**Figure 3:**
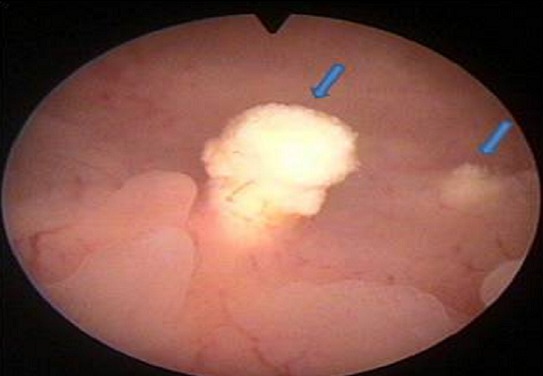
Image hystéroscopique montrant 2 fragments osseux à contours irréguliers partiellement couverts par l'endomètre (flèches)

**Figure 4:**
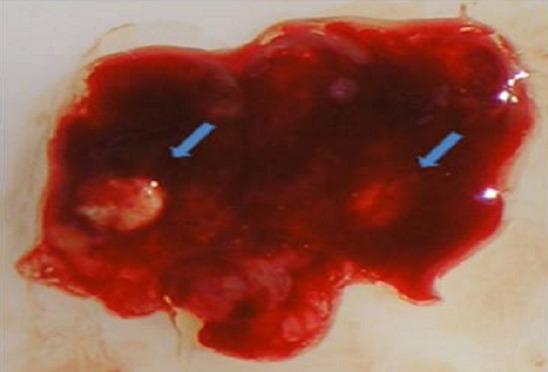
Aspect macroscopique du produit de curettage contenant les 2 fragments osseux (flèches)

**Figure 5:**
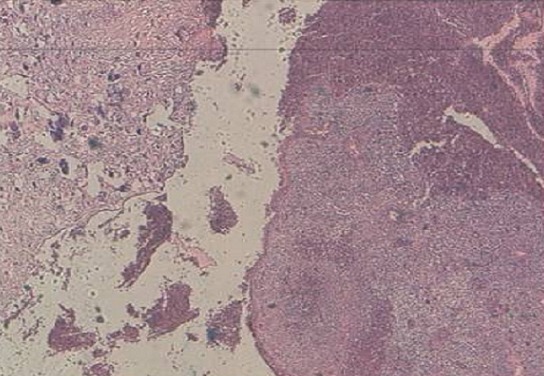
Histologie: Hes x 10: muqueuse endométriale largement dissociée par des remaniements calciques et fibreux

Mme B.F âgée de 43 ans, G3P 3 ayant présenté à j20 du post partum du dernier accouchement (ayant donné naissance à un nouveau-né âgé actuellement de 1an et demi) des métrorragies, pour lesquelles elle a été admise dans une autre structure et a bénéficié d'un curetage pour rétention placentaire. Elle a consulté dans notre formation pour des métrorragies associées à des algies pelviennes chronique et des leucorrhées jaunâtres fétides ayant débuté 2 mois après le curetage. L'examen gynécologique retrouve des leucorrhées blanchâtres. Un prélèvement bactériologique fait était stérile. L’échographie pelvienne a trouvé un utérus de taille normale, siège d'une image semblant intracavitaire hyperéchgène avec conne d'ombre postérieur mesurant 8/5mm (Figure 1). L'hystérosonographie a objectivé une image intracavitaire ayant les mêmes caractéristiques, le tout ayant évoqué un polype calcifié (Figure 2). Une hystéroscopie diagnostique est alors réalisée, Laquelle a trouvé au niveau de la paroi postérieur une petite formation décrite comme étant un polype calcifié juxtaposé à une autre lésion beaucoup plus petite, ayant les mêmes caractéristiques (Figure 3). Un curetage de l'endomètre a été réalisé (Figure 4) dont l’étude histologique a permis de poser le diagnostic de métaplasie ostéoïde de l'endomètre (Figure 5).

## Discussion

La MOE est une pathologie rare dont la fréquence a été estimée à 0,3 pour 1000 femmes infertiles [1, 2]. En effet, notre cas est le premier diagnostiqué dans notre formation, malgré que la prédisposition ethnique africaine a été évoquée dans la littérature [2, 3].

Classiquement, la MOE est diagnostiquée dans un contexte d'antécédent d'avortement surtout s'il est à répétition, tardif, ou s'il a fait l'objet d'une révision utérine instrumentale [2]. Le délai séparant l'avortement du diagnostic de la MOE étant très variable (quelques jours à plusieurs années [1]. A notre connaissance notre cas est le premier rapporté suite à un accouchement à terme. La MOE agirait comme un DIU et peut se traduire par une infertilité secondaire, en effet cette dernière constitue le signe clinique le plus fréquent [4], mais d'autres modes de révélation existent à type de dysménorrhées, leucorrhées, algies pelviennes chroniques en rapport avec un tableau d'endométrite chronique aseptique, et plus rarement l'expulsion spontanée de fragments osseux [1–5]. Cependant, des cas asymptomatiques ont été rapportés [2].

La physiopathologie de cette entité n'est toujours pas claire [6]. Plusieurs hypothèses ont été proposée à savoir une rétention in utero de fragments d'os fœtaux, une greffe endométriale spontanée ou provoquée par un coup de curette de cellules fœtales mésenchymateuses à potentiel osseux, une origine müllérienne embryologique (sous l'influence de traumatismes locaux) et la théorie la plus acceptée est le remplacement du chorion dans les suites d'un curetage ou d'une endométrite par un tissu conjonctif cicatriciel d'origine maternel qui verrait alors ses cellules mésenchymateuses se transformer en ostéoblastes [1, 2, 5]. En effet, certains auteurs ont rapporté quelque cas sporadiques de MOE chez des femmes nulligestes ayant consulté pour infertilité primaire, ce qui plaide en faveur de cette théorie [4]. De plus, l'origine maternelle a été confirmée par Enrique Cayuela grâce à l’étude d'ADN d'un cas de MOE [7]. Dans notre cas la MOE pourrait être expliqué par la transformation des cellules mésenchymateuses du chorion endométrial en ostéoblastes dans les suites du curetage que la patiente a subi en raison de la rétention hémorragique en post partum. Ainsi notre cas est en lui-même un argument de plus qui s'ajoute à celui d'Enrique Cayuela en faveur de l'origine maternelle de la MOE.

L’échographie montre typiquement une image hyperéchogène avec un cône d'ombre postérieur, des contours flous, souvent d'aspect linéaire, en situation intracavitaire [2–8], et persistante tout au long du cycle [1]. Cependant cette aspect peut prêter à confusion avec d'autres étiologies comme le polype endométrial calcifié - ce qui est le cas chez notre patiente- La tuberculose génitale, la métaplasie squameuse ou musculaire le fibrome calcifié, la calcification des artères du myomètre, la cicatrice stellaire post-césarienne ou postmyomectomie [9], les tumeurs malignes mullériennes, les tératomes ou enfin un corps étranger à type de DIU au cuivre [2].

A‘ ce jour, l'imagerie par résonance magnétique (IRM) ne présente pas d'intérêt dans ce type de pathologie [2] et l'hystérosonographie peut avoir un intérêt particulier quand le doute persiste sur la localisation intracavitaire ou pas de l'image, comme dans notre cas où elle a confirmé la situation intracavitaire.

L'hystéroscopie trouve des copeaux osseux irréguliers, dentelés, enchevêtrés, de couleur blanchâtre avec un aspect coralliforme, ou de coquille d'oeuf; des plaques d'ossification encastrées dans la partie profonde de l'endomètre au contact du myomètre et parfois même de petits os nettement reconnaissables (fémur, tibia, scapula...). Cependant, l'hystéroscopie peut s'avérer normale lorsque les fragments osseux sont enlisés profondément dans le myomètre et recouverts d'endomètre normal [2].

Le traitement optimale est basé sur l'hystéroscopie opératoire permettant l'ablation des fragments osseux à l'aide de l’énergie mécanique ou électrique mais avec grande précaution (mesure de l’épaisseur myométriale séparant les fragments osseux de la séreuse utérine [2] et pour certains un guidage échographique) [1, 2, 4, 5] vue le risque de perforation utérine. L'ablation de tous les fragments est nécessaire afin d'obtenir une vacuité utérine, ce qui permet de traiter la possible endométrite chronique et même d'avoir une grossesse dans certains cas d'infertilité [5]. Par ailleurs le curetage devrait être bannit puis qu'il ne permet pas un traitement complet et qu'il est très traumatique pour l'endomètre et donc plus fréquemment source de synéchies chez des patientes souvent jeune et désireuse de procréation [2]. L’étude histologique est d'un grand apport diagnostic en permettant notamment d'exclure d'autres pathologies telles les tumeurs mulleriennes mixtes de l'utérus dont le traitement est différent de celui proposé pour la MOE [4].

A distance du traitement, Le risque de récidive existe mais il est difficile à estimer, incitant au suivi clinique, échographique voire hystéroscopique de ces patientes [1]. Dans notre cas, le contrôle clinique 6 mois après le diagnostic a trouvé une patiente asymptomatique, et un contrôle échographique est prévu 6 mois plus tard.

## Conclusion

L'attention du gynécologue devrait être attiré par les images hyperéchogènes (en cas d'infertilité secondaire, après une grossesse arrêtée ou une IVG à des termes avancées avec gestes endo-utérins) surtout dans notre contexte, puisque les femmes africaines semblent être plus prédisposées à la MOE. L'hystéroscopie doit alors être réalisée et le curetage de l'endomètre devrait être bannit dans ces cas puisque la résection élective des foyers de MOE reste le traitement de choix avec un pronostic favorable sur la fertilité ultérieure.
